# Diagnostic performance of magnifying endoscopy with third-generation narrow-band imaging for early gastric cancer: post hoc analysis of a randomized trial (3G detection trial)

**DOI:** 10.1007/s00535-026-02434-0

**Published:** 2026-05-14

**Authors:** Nobuhisa Minakata, Tomohiro Kadota, Seiichiro Abe, Noriya Uedo, Hisashi Doyama, Yasuaki Furue, Akira Yokoyama, Satoru Nonaka, Yasuhiro Tani, Naohiro Yoshida, Chikatoshi Katada, Manabu Muto, Takashi Ikeno, Masashi Wakabayashi, Tomonori Yano

**Affiliations:** 1 Department of Gastroenterology and Endoscopy, National Cancer Center Hospital East, 6-5-1, Kashiwanoha, Kashiwa, Chiba 277-8577 Japan; 2 Endoscopy Division, National Cancer Center Hospital, Tokyo, Japan; 3 Department of Gastrointestinal Oncology, Osaka International Cancer Institute, Osaka, Japan; 4 Department of Gastroenterology, Ishikawa Prefectural Central Hospital, Ishikawa, Japan; 5 Department of Gastroenterology, Kitasato University School of Medicine, Kanagawa, Japan; 6 Department of Therapeutic Oncology, Kyoto University Graduate School of Medicine, Kyoto, Japan; 7 Clinical Research Support Office, National Cancer Center Hospital East, Kashiwa, Japan; 8 Biostatistics Division, Center for Research Administration and Support, National Cancer Center Hospital East, Kashiwa, Japan

**Keywords:** Diagnosis, Gastric cancer, Gastrointestinal endoscopy, Narrow band imaging

## Abstract

**Background:**

In this study, we investigated the diagnostic performance of magnifying endoscopy (ME) with third-generation narrow-band imaging (3G-NBI) for early gastric cancer (EGC), primarily in patients with *Helicobacter pylori* eradication.

**Methods:**

This was a post hoc analysis of a multicenter, randomized trial comparing 3G-NBI, texture and color enhancement imaging, and white-light imaging in gastric neoplasm (GN) detection. For all detected lesions, the endoscopic diagnosis of ME using 3G-NBI was compared with the pathological diagnosis. The primary analyses focused on the sensitivity and specificity of ME with 3G-NBI for EGC or non-EGC. The diagnostic performance was analyzed according to confidence level, macroscopic type, lesion size, and *H. pylori* infection status.

**Results:**

This study included 901 patients; 228 suspected GN lesions in 187 patients were analyzed. The lesions were diagnosed with EGC in 62 (27 with high confidence) and non-EGC in 166 (91 with high confidence) patients using ME with 3G-NBI and pathologically diagnosed as EGC in 61 and non-EGC in 167 patients. The overall diagnostic performance was sensitivity and specificity of 70.5% and 88.6%, respectively. The diagnostic performance of each category was as follows: (1) confidence level (high/low); sensitivity 78.1%/62.1%, specificity 97.7%/79.0%; (2) macroscopic type (elevated/flat or depressed); sensitivity 84.6%/66.7%, specificity 96.7%/86.9%; (3) lesion diameter (< 10 mm/ ≥ 10 mm); sensitivity 65.7%/76.9%, specificity 88.6%/88.9%; (4) *H. pylori* infection status (uninfected/previously infected/currently infected); sensitivity 50.0%/71.0%/86.7%, specificity 91.3%/88.2%/88.9%.

**Conclusions:**

The diagnostic performance of ME with 3G-NBI for EGC was acceptable, primarily in patients with *H. pylori* eradication.

**Trial Registration:**

This trial was registered in jRCT (Identifier jRCT1032210213).

**Supplementary Information:**

The online version contains supplementary material available at 10.1007/s00535-026-02434-0.

## Introduction

Gastric cancer (GC) has the fifth highest incidence and mortality rates among all cancer types worldwide [1]. GC is often diagnosed at an advanced stage, and its prognosis remains poor [2]. Therefore, early detection of GC is important to improve prognosis. Therefore, endoscopic screening has traditionally been used for the early detection of GC and precancerous lesions, such as gastric adenomas, in Korea and Japan, where GC incidence is high. Recently, endoscopic screening was found to reduce the risk of death from GC [3–5].

Generally, when lesions suspected to be early gastric neoplasms (GNs), including early gastric cancers (EGCs) and gastric adenomas, are detected during endoscopic screening, they are endoscopically diagnosed as EGC or non-EGC, and biopsy is performed if necessary. To endoscopically differentiate between EGC and non-EGC, magnifying endoscopy (ME) with narrow-band imaging (NBI) is used, which can identify a demarcation line (DL) between the lesion and the background mucosa and visualize microvascular (MV) and microsurface (MS) patterns, with a reported sensitivity of 60.0%–60.7% and specificity of 93.1%–98.0% [6–9].

Previous studies have focused on the diagnostic performance of ME with NBI in populations mostly infected with *Helicobacter pylori* [6–9]. In the past, many people in Japan were infected with *H. pylori*, but as *H. pylori* infection has been found to be a risk factor for GC development, *H. pylori* eradication treatment to prevent heterochronic EGC was covered by insurance in 2013, and patients who have undergone *H. pylori* eradication are currently increasing [10, 11]. EGC detected after *H. pylori* eradication often shows gastritis-like features owing to non-neoplastic epithelial covering of the cancerous tissue, which may be difficult to diagnose correctly using ME with NBI [12–14]. Thus, the diagnostic performance of ME with NBI for EGC or non-EGC varies significantly depending on *H. pylori* infection status. However, the diagnostic performance in a population with *H. pylori* eradication is unknown.

Furthermore, these reports examined the diagnostic performance of ME in previous generations of NBI [6, 7, 15–17]. The latest endoscopy system, CV1500 (Olympus Medical Systems Corp., Tokyo, Japan), features improved white-light imaging (WLI) and third-generation NBI (3G-NBI), including reduced noise, less color shift, brighter and higher image quality, as well as texture and color enhancement imaging (TXI) as a new image-enhanced endoscopy (IEE) [18]. ME with 3G-NBI is expected to further facilitate the differentiation between EGC and non-EGC. Recently, we reported a multicenter, randomized, open-label, three-arm-parallel phase II trial comparing 3G-NBI, TXI, and WLI for GN detection [19]. Patients with *H. pylori* eradication were mainly included, and the protocol stipulated that all lesions suspected to be GN were endoscopically diagnosed as EGC or non-EGC using ME with 3G-NBI and pathologically diagnosed by biopsy.

Using data from the previous phase II study, we aimed to investigate the diagnostic performance of ME with the latest generation NBI for EGC or non-EGC in a population primarily comprising patients with *H. pylori* eradication.

## Methods

### Summary of a randomized three-arm phase II trial

The main trial was a multicenter, randomized study to determine the optimal imaging modality for identifying new GNs in high-risk patients using WLI, 3G-NBI, and TXI. Patients were enrolled from August 2021 to June 2022. Key eligibility criteria included scheduled surveillance endoscopy after endoscopic resection (ER) for GN or ER, chemotherapy or radiotherapy for esophageal cancer, or preoperative endoscopy for known GN or esophageal cancer [19–21]. Patients were randomly assigned in a 1:1:1 ratio to WLI (primary and secondary WLI), 3G-NBI (primary 3G-NBI and secondary WLI), and TXI (primary TXI and secondary WLI) group.

The examination protocol comprised non-ME observations, including primary and secondary observations, ME with 3G-NBI, and biopsies if target lesions were found. Primary observation of the entire stomach was performed to detect GN. Subsequently, secondary WLI was immediately performed by the same endoscopist to detect any missed GNs. Target lesions, defined as newly detected lesions suspected to be GN by non-ME, had endoscopic EGC or adenoma characteristics, such as mucosal discoloration or morphological changes in the mucosal surface [16, 19]. Lesions with findings typical of advanced GC and pre-existing lesions were not considered target lesions. If the target lesion was detected, detailed magnification with 3G-NBI was subsequently performed to differentiate between EGC and non-EGC, as described below. To obtain a pathological diagnosis, all target lesions were biopsied at the end of the examination regardless of the endoscopic diagnosis. Subsequent treatments for the target lesions, such as endoscopic or surgical resection, were performed as necessary.

### Study design

This was a post hoc analysis in a randomized controlled trial (RCT) [19].

### Endoscopic systems

A single-channel upper gastrointestinal magnifying endoscope (GIF-XZ1200; Olympus Medical Systems Corp., Tokyo, Japan) equipped with an endoscopic system (CV1500; Olympus Medical Systems Corp.) was used. The video processor settings for structural enhancement were type B, level 4 or 6 for WLI; type B, level 8 for 3G-NBI; and hard enhancement for TXI.

### Diagnosis of EGC using ME with 3G-NBI

For all target lesions, endoscopic diagnosis of EGC or non-EGC was performed using the Magnifying Endoscopy Simple Diagnostic Algorithm of EGC (MESDA-G) using ME with 3G-NBI [15, 16]. If the target lesion is detected, DL between the lesion and the background mucosa should be identified by ME with 3G-NBI; if a DL is absent, the lesion can be diagnosed as benign. If DL is present, the presence of an irregular MV and MS pattern should be determined. EGC can be diagnosed if irregular MV and/or MS patterns are present within the boundaries.

**Fig. 1:**
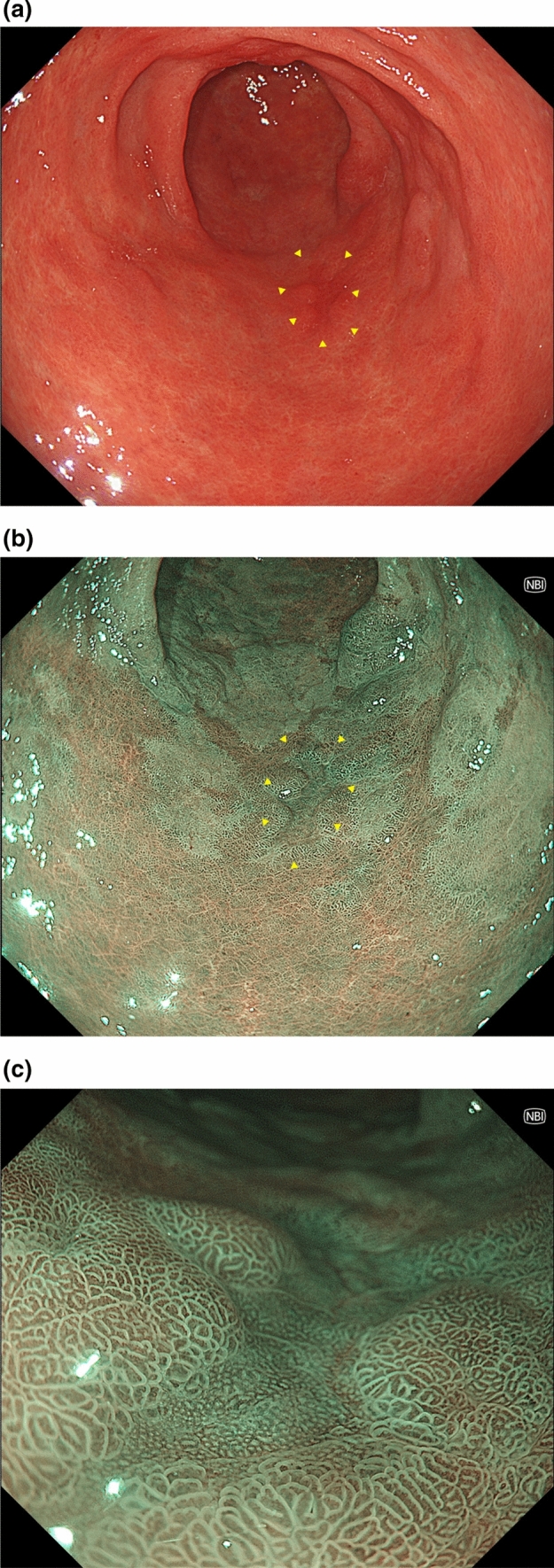
Representative images of the lesion diagnosed as non-EGC with high confidence in patients with *H. pylori* eradication. A depressed lesion in the lower third of the stomach is indicated (yellow arrows). The final histopathological diagnosis did not indicate gastric neoplasia, including gastric adenoma. **A** On WLI, the lesion appears as a reddish area with clear margins. **B** 3G-NBI shows the lesion as a brownish area. **C** Magnifying endoscopy with 3G-NBI showing the absence of irregular microvascular and microsurface patterns with a demarcation line. Therefore, the lesion was diagnosed as non-EGC with a high confidence level. EGC, early gastric cancer; WLI, white-light imaging; 3G-NBI, third-generation narrow-band imaging

**Fig. 2:**
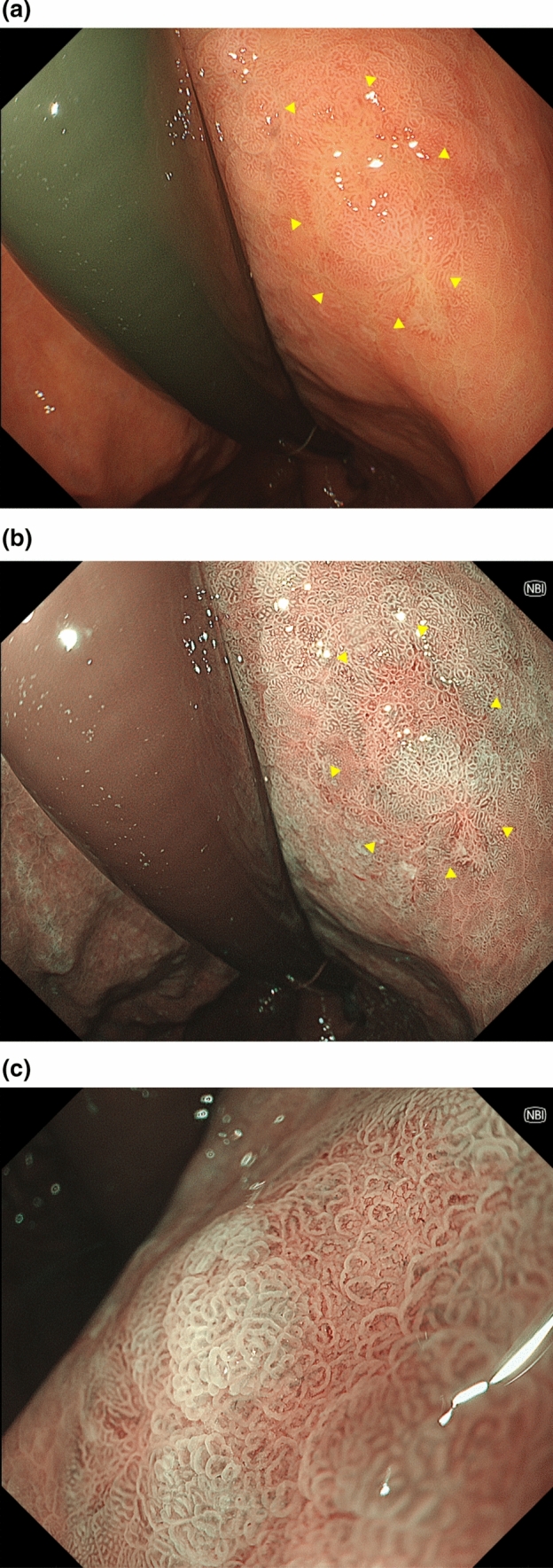
Representative images of the lesion diagnosed as EGC with high confidence in patients with *H. pylori* eradication. A depressed lesion in the middle third of the stomach is indicated (yellow arrows). The final histopathological diagnosis was well-differentiated tubular adenocarcinoma. **A** On WLI, the lesion appeared as a reddish area. **B** 3G-NBI shows the lesion as a brownish area. **C** Magnifying endoscopy with 3G-NBI showing the presence of irregular microvascular and microsurface patterns with a demarcation line. Therefore, the lesion was diagnosed as EGC with a high confidence level. EGC, early gastric cancer; WLI, white-light imaging; 3G-NBI, third-generation narrow-band imaging

**Fig. 3:**
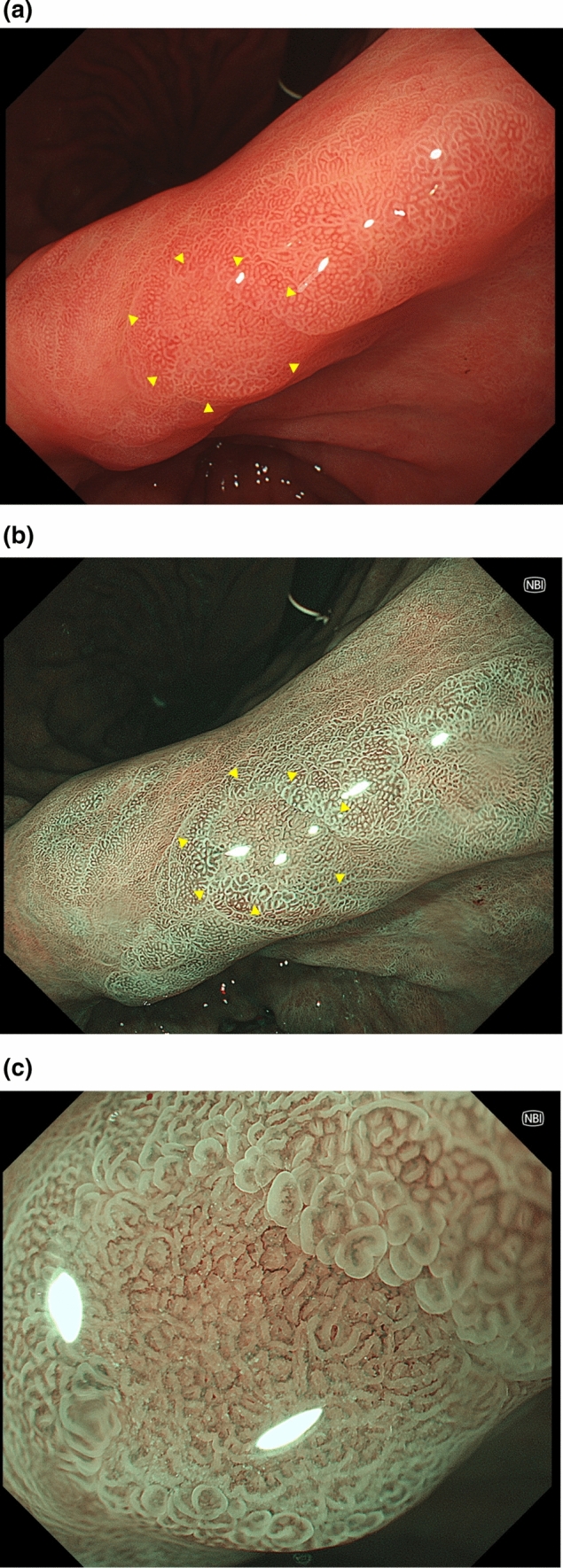
Representative images of the lesion diagnosed as non-EGC with low confidence in patients with *H. pylori* eradication. A depressed lesion in the lower third of the stomach is indicated (yellow arrows). The final histopathological diagnosis did not indicate gastric neoplasia, including gastric adenoma. **A** On WLI, the lesion appears as a reddish area with clear margins. **B** 3G-NBI shows the lesion as a brownish area. **C** Magnifying endoscopy with 3G-NBI clearly shows a demarcation line, but it is difficult to determine whether irregular microvascular and microsurface patterns are present, and the confidence level is indeterminate. Therefore, the lesion was diagnosed as non-EGC with a low confidence level. EGC, early gastric cancer; WLI, white-light imaging; 3G-NBI, third-generation narrow-band imaging

The examiner recorded the confidence level (Grades 1–5) of the endoscopic diagnosis when diagnosing EGC or non-EGC using ME with 3G-NBI: Grade 1, non-cancer with high confidence (The lesion can be diagnosed as non-cancer from the endoscopic findings alone. Biopsies of the lesion do not need to be taken under normal conditions.); Grade 2, suspected non-cancer with low confidence (The lesion has the appearance of non-cancer from the endoscopic findings. However, biopsies need to be taken from the lesion to confirm the diagnosis.); Grade 3, indeterminate (The lesion is indeterminate for non-cancer or cancer from the endoscopic findings alone. Therefore, biopsies need to be taken from the lesion to make a definitive diagnosis.); Grade 4, suspected cancer with low confidence (The lesion is suspicious for cancer from the endoscopic findings. However, biopsies need to be taken from the lesion to confirm the diagnosis.); and Grade 5, cancer with high confidence (The lesion can be diagnosed as cancer from the endoscopic findings alone. Biopsies of the lesion do not need to be taken under normal conditions.), similar to a previous report [6, 8]. Furthermore, Grades 1–3 were reclassified as non-EGC and Grades 4 and 5 as EGC. Representative images are shown in Figs. 1, 2, 3. To maintain endoscopic quality control, the study protocol prespecified that all examinations be performed by endoscopists who were board-certified fellows of the Japan Gastroenterological Endoscopy Society or who had equivalent qualifications, as described in the main study [19]. In total, 37 endoscopists participated in this post hoc study. All participating endoscopists routinely used ME with 3G-NBI for EGC assessment in daily clinical practice. In addition, before study initiation, they were trained using WLI, 3G-NBI, and TXI endoscopic images of gastric lesions, and all IEEs were used for stomach observation training, as reported in the primary study [19].

### Pathological evaluation

Pathological diagnoses were made by expert pathologists at each institution based on biopsied tissues or specimens obtained via endoscopic or surgical resection. If both biopsied tissues and resected specimens were available, the final diagnosis was determined based on the more important diagnosis. Differentiation among EGC, gastric adenoma, and non-neoplasm was based on the Japanese Classification of Gastric Cancer [22] and the revised Vienna classification [23]. Specifically, high-grade dysplasia, in accordance with the WHO classification, corresponds to mucosal adenocarcinoma according to the Japanese Classification of Gastric Cancer [22, 24]. When gastric adenomas are assessed endoscopically using the MESDA-G algorithm, they frequently exhibit regular MS and MV patterns and are, therefore, classified as non-EGC [25–27]. To ensure consistency between the endoscopic and pathological diagnoses, pathologically confirmed gastric adenomas were classified as non-EGC in this study.

### Statistical analysis

The primary analyses were the sensitivity and specificity of ME with 3G-NBI for EGC or non-EGC, and the secondary analyses were the proportion of accurate diagnosis (accuracy), positive predictive value (PPV), and negative predictive value (NPV). Diagnostic performance was evaluated after excluding pathological gastric adenomas. Furthermore, the diagnostic performance was analyzed according to confidence level (high; Grade 1 and 5/low; Grade 2–4), macroscopic type (elevated/flat or depressed), lesion size (< 10 mm/≥ 10 mm), and *H. pylori* infection status (uninfected/previously infected/currently infected). The *H. pylori* infection status was determined on the basis of patient interviews, endoscopic findings, and *H. pylori* testing. If a history of eradication therapy was confirmed, the *H. pylori* infection status was determined to be “previously infected.” If there was no history of eradication therapy and positive results were obtained in *H. pylori* testing, the patient was considered “currently infected.” If the test results were negative, the patient was basically considered “uninfected.” If there was no history of *H. pylori* testing or the outcome after eradication therapy had not yet been determined, the infection status was considered “unknown” [28].

For the diagnosis of EGC or non-EGC, the endoscopic diagnosis was compared with pathological diagnosis using Fisher’s exact test, and the sensitivity, specificity, accuracy, PPV, and NPV with their 95% confidence intervals, were calculated. All p-values were two-sided with a significance level of 0.05.

All statistical analyses were performed using SAS (version 9.4; Cary, NC, USA).

## Results

### Background characteristics

**Fig. 4:**
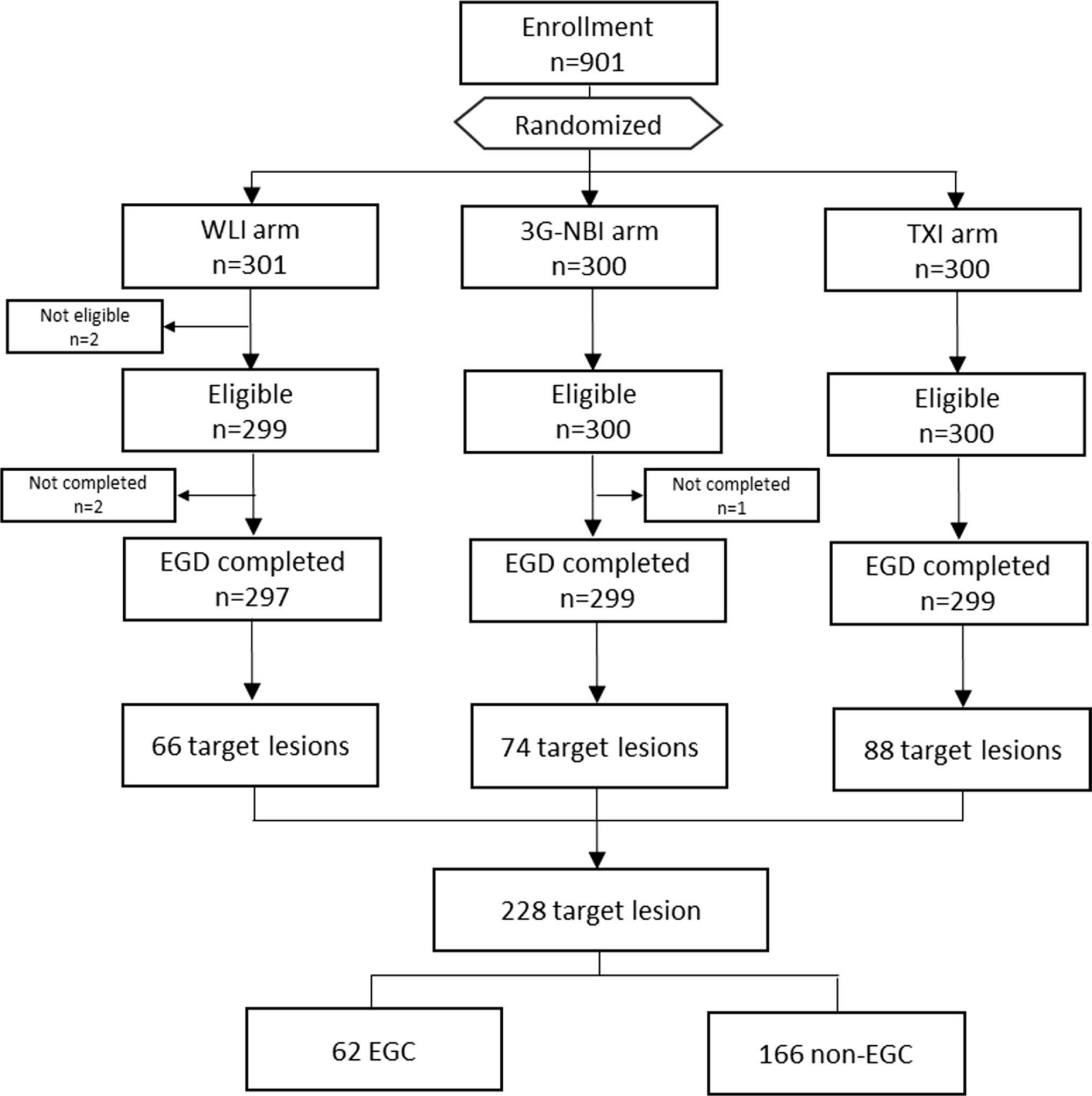
Patient flow diagram. WLI, white-light imaging; 3G-NBI, third-generation narrow-band imaging; TXI, texture and color enhancement imaging; EGD, esophagogastroduodenoscopy; EGC, early gastric cancer

Of the 901 patients enrolled in the previously published phase II trial, baseline characteristics were similar between the 3G-NBI (*n* = 300), TXI (*n* = 300), and WLI (*n* = 301) groups [19]. In the 3G-NBI group, 63 and 11 target lesions were detected during primary 3G-NBI and secondary WLI observations, respectively; in the TXI group, 75 and 13 were detected during primary TXI and secondary WLI observations, respectively; and in the WLI group, 57 and 9 were detected during primary and secondary WLI observations (Fig. 4). Overall, 228 target lesions from 187 patients were analyzed.

As shown in Table 1, 43 lesions (18.9%) were elevated, and 185 (81.1%) were flat or depressed. Lesions < 10 mm were 166, lesions ≥ 10 mm were 62, and the median size of the lesions was 5.0 mm (range: 1.0–40.0). Thirty-two lesions (14.0%) were located in the upper third of the stomach, 44 (19.3%) in the middle third, and 152 (66.7%) in the lower third. *H. pylori* infection status was as follows: 124 (54.4%) were previously infected, 27 (11.8%) were uninfected, 42 (18.4%) were currently infected, and 35 (15.4%) had unknown infection status. Flat or depressed lesions accounted for 84.7% (105/124) of the previously infected group, 77.8% (21/27) of the currently infected group, and 76.2% (32/42) of the uninfected group (Online Resource 1).

**Table 1:** Patient and lesion characteristics

Patient characteristics		
Age (years), median (range)	74	(35–85)
Sex, male, *n* (%)	144	(77.0)
Lesion characteristics		
Size (mm)		
Median (range)	5.0	(1.0–40.0)
< 10 mm, *n* (%)	166	(72.8)
≥ 10 mm, *n* (%)	62	(27.2)
Location, *n* (%)		
Upper third	32	(14.0)
Anterior wall	4	(1.8)
Lesser curvature	11	(4.8)
Posterior wall	14	(6.1)
Greater curvature	3	(1.3)
Middle third	44	(19.3)
Anterior wall	5	(2.2)
Lesser curvature	20	(8.8)
Posterior wall	12	(5.3)
Greater curvature	7	(3.1)
Lower third	152	(66.7)
Anterior wall	30	(13.2)
Lesser curvature	45	(19.7)
Posterior wall	35	(15.4)
Greater curvature	42	(18.4)
Macroscopic type, *n* (%)		
Elevated	43	(18.9)
Flat or depressed	185	(81.1)
Endoscopic diagnosis, *n* (%)		
EGC	62	(27.2)
High confidence	27	(11.8)
Low confidence	35	(15.4)
Non-EGC	166	(72.8)
High confidence	91	(39.9)
Low confidence	75	(32.9)
Pathological diagnosis, *n* (%)		
EGC	61	(26.8)
Differentiated type	48	(21.1)
Undifferentiated type	3	(1.3)
Unknown	10	(4.4)
Non-EGC	167	(73.2)
Negative for neoplasm	159	(69.7)
Gastric adenoma	8	(3.5)
* H. pylori* infection status, *n* (%)		
Previously infected	124	(54.4)
Uninfected	27	(11.8)
Currently infected	42	(18.4)
Unknown	35	(15.4)

*EGC* early gastric cancer

### Overall diagnostic performance of ME with 3G-NBI

Using ME with 3G-NBI, 62 of 228 target lesions (27.2%) were diagnosed as EGC, of which 27 were diagnosed with high confidence, whereas 166 (72.8%) were diagnosed as non-EGC, of which 91 were diagnosed with high confidence. Finally, 61 (26.8%) were pathologically diagnosed with EGC and 167 (73.2%) were pathologically diagnosed with non-EGC, of which 8 were gastric adenomas. ME with 3G-NBI for EGC showed a sensitivity of 70.5% (43/61), specificity of 88.6% (148/167), accuracy of 83.8% (191/228), PPV of 69.6% (43/62), and NPV of 89.2% (148/166) (Table 2 and Online Resource 2). When gastric adenomas were excluded, ME with 3G-NBI for EGC showed a sensitivity of 70.5% (43/61), specificity of 90.0% (143/159), accuracy of 84.5% (186/220), PPV of 72.9% (43/59), and NPV of 88.8% (143/161) (Online Resource 3 and 4).

**Table 2:** Overall diagnosis performance of ME with 3G-NBI

A		Pathological diagnosis, *n*	
		EGC	Non-EGC
Endoscopic diagnosis	EGC	43	19
	Non-EGC	18	148

B			
Sensitivity, % (*n*/*n*) (95% CI)	70.5	(43/61)	(57.4–81.5)
Specificity, % (n/n) (95% CI)	88.6	(148/167)	(82.8–93.0)
PPV, % (*n*/*n*) (95% CI)	69.4	(43/62)	(56.4–80.4)
NPV, % (*n*/*n*) (95% CI)	89.2	(148/166)	(83.4–93.5)
Accuracy, % (*n*/*n*) (95% CI)	83.8	(191/228)	(78.3–88.3)

*ME* magnifying endoscopy, *3G*-*NBI* third-generation narrow-band imaging, *EGC* early gastric cancer, *PPV* positive predictive value, *NPV* negative predictive value, *CI* confidence interval

### Subgroup analysis

Based on the confidence level in endoscopic diagnosis, of 27 lesions that were endoscopically diagnosed as EGC with high confidence, 25 (92.6%) were pathologically diagnosed as EGC. Of 91 endoscopically diagnosed as non-EGCs with high confidence, 84 (92.3%) were pathologically diagnosed as non-EGCs (Online Resource 2). For the lesions diagnosed with high and low confidence, sensitivities were 78.1% (25/32) and 62.1% (18/29) (*p* = 0.261), and specificities were 97.7% (84/86) and 79.0% (64/81) (*p* < 0.001), respectively. According to the macroscopic type, sensitivity was 84.6% (11/13) and 66.7% (32/48), and specificity was 96.7% (29/30) and 86.9% (119/137) for elevated lesions and flat or depressed lesions, respectively. According to lesion size, sensitivity was 65.7% (23/35) and 76.9% (20/26), and specificity was 88.6% (116/131) and 88.9% (32/36) in the lesions measuring < 10 mm and ≥ 10 mm, respectively. As for *H. pylori* infection status, sensitivity was 50.0% (2/4), 71.0% (22/31), and 86.7% (13/15), and specificity was 91.3% (21/23), 88.2% (82/93), and 88.9% (24/27) in the *H. pylori* uninfected, previously infected, and currently infected group, respectively (Table 3).

**Table 3:** Results of subgroup analysis

	Sensitivity, % (*n*/*n*) (95% CI)			Specificity, % (*n*/*n*) (95% CI)			PPV, % (*n*/*n*) (95% CI)			NPV, % (*n*/*n*) (95% CI)			Accuracy, % (*n*/*n*) (95% CI)		
**Confidence level**															
High confidence	78.1	(25/32)	(60.0–90.7)	97.7^***^	(84/86)	(91.9–99.7)	92.6^***^	(25/27)	(75.7–99.1)	92.3	(84/91)	(84.8–96.9)	92.4^***^	(109/118)	(86.0–96.5)
Low confidence	62.1	(18/29)	(42.3–79.3)	79.0	(64/81)	(68.5–87.3)	51.4	(18/35)	(34.0–68.6)	85.3	(64/75)	(75.3–92.4)	74.6	(82/110)	(65.4–82.4)
**Macroscopic type**															
Elevated	84.6	(11/13)	(54.6–98.1)	96.7	(29/30)	(82.8–99.9)	91.7	(11/12)	(61.5–99.8)	93.6	(29/31)	(78.6–99.2)	93.0	(40/43)	(80.9–98.5)
Flat or depressed	66.7	(32/48)	(51.6–78.6)	86.9	(119/137)	(80.0–92.0)	64.0	(32/50)	(49.2–77.1)	88.2	(119/135)	(81.5–93.1)	81.6	(151/185)	(75.3–86.9)
**Lesion size**															
< 10 mm	65.7	(23/35)	(47.8–80.9)	88.6	(116/131)	(81.8–93.4)	60.5	(23/38)	(43.4–76.0)	90.6	(116/128)	(84.2–95.1)	83.7	(139/166)	(77.2–89.0)
≥ 10 mm	76.9	(20/26)	(65.4–91.0)	88.9	(32/36)	(73.9–96.9)	83.3	(20/24)	(62.6–95.3)	84.2	(32/38)	(68.8–94.0)	83.9	(52/62)	(72.3–92.0)
***H. pylori* infection status**															
Previously infected	71.0	(22/31)	(52.0–85.8)	88.2	(82/93)	(79.8–93.9)	66.7	(22/33)	(48.2–82.0)	90.1	(82/91)	(82.1–95.4)	83.9	(104/124)	(76.2–89.9)
Uninfected	50.0	(2/4)	(6.8–93.2)	91.3	(21/23)	(72.0–98.9)	50.0	(2/4)	(6.8–98.9)	91.3	(21/23)	(72.0–98.9)	85.2	(23/27)	(66.3–95.8)
Currently infected	86.7	(13/15)	(59.5–98.3)	88.9	(24/27)	(70.8–97.7)	81.3	(13/16)	(54.4–96.0)	92.3	(24/26)	(74.9–99.1)	88.1	(37/42)	(74.4–96.0)

^***^ P < 0.001, no significant differences in other factors

*ME* magnifying endoscopy, *3G*-*NBI* third-generation narrow-band imaging, *PPV* positive predictive value, *NPV* negative predictive value

Focusing on the population of combinations for each group, the diagnostic accuracy of ME with 3G-NBI was high in elevated lesions regardless of confidence level (high: 90.9% [20/22], low: 95.2% [20/21]), whereas it was significantly lower with low confidence level (69.7% [62/89]) than with high confidence level (92.7% [89/96]) in flat or depressed lesions (*p* < 0.001). In particular, in the previously infected group, there was a difference in the diagnostic accuracy of ME with 3G-NBI for flat or depressed lesions between confidence levels (high: 91.7% [55/60] vs low: 66.7% [30/45], *p* = 0.0019) (Online Resource 5).

## Discussion

To our knowledge, this is the first study to examine the diagnostic performance of ME with 3G-NBI for EGC in a large number of cases using multicenter, prospectively collected data. No other study has examined this diagnostic performance in a population primarily comprising patients with *H. pylori* eradication. Moreover, regarding the overall diagnostic performance of ME with 3G-NBI for EGC, the sensitivity was good, but the specificity was insufficient. A subgroup analysis revealed that in elevated lesions or lesions diagnosed with high confidence, the diagnostic performance of ME with 3G-NBI for EGC was favorable. However, for smaller lesions or flat or depressed lesions, the diagnostic performance was not satisfactory.

Previous studies reported a sensitivity of 60.0%–60.7% and specificity of 93.1%–98.0% for ME with NBI; compared with these studies, our study showed a tendency for improved sensitivity and worsened specificity [6–9].

Kakushima et al. reported the diagnostic performance of ME using second-generation NBI (2G-NBI) as a secondary analysis in a phase III study [6]. Compared with the findings in this report, for elevated lesions, the diagnostic performance of ME with 3G-NBI for EGC improved (sensitivity: 84.6% vs. 60.6%, specificity: 96.7% vs. 88.8%). In addition, although the percentage of lesions diagnosed with high confidence by ME with 3G-NBI was higher than that with 2G-NBI (51.8% vs 38.3%), for lesions diagnosed with high confidence, the sensitivity and specificity of ME with 3G-NBI for EGC were comparable with those of 2G-NBI (sensitivity: 78.1% vs. 64.7%, specificity: 97.7% vs. 97.2%). The improved diagnostic performance is presumably due to the benefit of clear images with 3G-NBI, which makes it easier to differentiate EGCs from other elevated lesions, such as gastric polyps and adenomas in elevated lesions; identifying DL and recognizing MS and MV patterns in lesions with high confidence is also easier, resulting in a more confident cancer or non-cancer decision [29–31]. Conversely, in this study, lesions < 10 mm had sensitivity and specificity comparable to those for lesions > 10 mm, although PPV tended to be lower (PPV; 60.5% vs. 83.3%, *p* = 0.089), probably because smaller do not benefit from clear images with 3G-NBI because of the limited area to be evaluated.

In flat or depressed lesions, the sensitivity of ME with 3G-NBI was comparable, although its specificity was worse than that of 2G-NBI (sensitivity: 66.7% vs 60.7%; specificity: 86.9% vs 94.1%). The presence of benign or malignant flat or depressed lesions that are difficult to distinguish as EGC or non-EGC, given that our study population primarily comprised patients with *H. pylori* eradication, may have reduced the sensitivity and specificity. Benign flat or depressed lesions are erythematous flat or depression called “Mottled Patchy Erythema” or “map-like redness”, which may appear in the stomach following eradication of *H. pylori* [32, 33]. This can be difficult to differentiate from EGC because of histological alterations in the surface structures, including multiple appearances, which may have resulted in its frequent misdiagnosis as EGC even using ME with NBI [34, 35]. Conversely, EGCs resembling benign flat or depressed lesions can appear in the stomach following the eradication of *H. pylori*. Kobayashi et al. reported that “gastritis-like” EGCs, often recognized as an erythematous flat or depressed, that resemble the adjacent non-cancerous mucosa and display unclear demarcation occur more frequently in the successful *H. pylori* eradication group than the non-eradicated group [14]. Actually, in this study, the diagnostic accuracy of ME with 3G-NBI was high regardless of the confidence level in elevated lesions, whereas it was significantly lower in flat or depressed lesions diagnosed with a low confidence level compared to a high confidence level. Flat or depressed lesions diagnosed with low confidence in the previously infected group had the lowest diagnostic accuracy in the analysis. The results indicate the difficulty in differentiating EGC from non-EGC in flat or depressed lesions diagnosed with low confidence, and this difficulty may be related to previous *H. pylori* infection.

We evaluated the diagnostic performance of ME with 3G-NBI using a simplified binary classification of EGC versus non-EGC based on the MESDA-G algorithm. Although the endoscopic classification of gastric adenomas as either cancer or non-cancer can sometimes be challenging, gastric adenomas were included in the non-EGC group to maintain consistency with previously published studies [6, 8] and to facilitate direct comparison with them. Endoscopic misclassification of gastric adenomas as EGC could increase false-positive diagnoses and thereby lead to a reduction in specificity. However, previous reports have shown that most gastric adenomas exhibit regular MS and MV patterns on ME with NBI, which are useful for differentiating them from EGC [25–27]. Therefore, when the MESDA-G algorithm is applied, pathological gastric adenomas are expected to be frequently diagnosed endoscopically as non-EGC, and inclusion of gastric adenomas in the non-EGC group appears reasonable. Furthermore, among the 228 target lesions included in this study, only eight (3.5%) were gastric adenomas. As shown in Online Resources 3 and 4, the diagnostic performance excluding gastric adenomas was not substantially different from that including gastric adenomas, suggesting that the impact of classifying gastric adenomas as non-EGC on overall diagnostic accuracy was limited.

This study had some limitations. First, the interobserver agreement for the same lesion in the diagnosis of ME with 3G-NBI was not evaluated. Therefore, the reproducibility of diagnostic categorization and diagnostic confidence assessment remains uncertain. Nevertheless, since many endoscopists from 13 institutions participated in the protocol examination, this study’s results are valid compared to those of previous reports on the diagnostic performance of ME with NBI [6–9]. Second, the magnification rate was not clearly specified. Consequently, in some cases, the degree of magnification may not have been sufficient to allow the characterization of lesions as EGC or non-EGC, which could have potentially influenced the diagnostic performance. Despite this limitation, the diagnostic performance observed in this study remains consistent with previously reported outcomes, suggesting that the findings are meaningful [6–9]. Third, the pathology of the unresected lesions did not confirm the possibility of a discordance between biopsy and resected tissue. Most detected EGCs were resected; however, 10 GC cases were not resected for several reasons, including adverse events and patient refusal. Therefore, there is a possible discrepancy between the biopsy histology and primary histology of these lesions. Fourth, the sample size was relatively small, particularly for subgroup analyses, which limited statistical power and may have precluded the detection of significant differences in certain subgroups. Larger prospective studies are therefore required to validate these findings. To address these limitations, we are currently conducting a jRCT (Identifier jRCT1032230613) with larger sample sizes, and we look forward to the results.

In conclusion, advances in endoscopic instrumentation have facilitated EGC or non-EGC diagnosis with high confidence using ME with NBI. Although the endoscopic diagnosis of EGC or non-EGC using ME with NBI presents new challenges, particularly because of lesion characteristics in populations primarily comprising patients with *H. pylori* eradication, ongoing technological progress and clinical experience accumulation hold promise for overcoming these difficulties and achieving more accurate diagnoses.

## Supplementary Information

Below is the link to the electronic supplementary material.

Supplementary file1 (DOCX 23 KB)
