# Supplementary material for: Diagnostic performance of magnifying endoscopy with third-generation narrow-band imaging for early gastric cancer: post hoc analysis of a randomized trial (3G detection trial)
Source: J Gastroenterol. 2026 May 14;61(8):1093–103. doi: 10.1007/s00535-026-02434-0 (PMC13407715; doi:10.1007/s00535-026-02434-0)
Supplement: Supplementary file 1 — Supplementary file1 (DOCX 23 KB) [file 535_2026_2434_MOESM1_ESM.docx]

**Online Resource 1.** Relationship between macroscopic type and *H pylori* infection status

|  | ***H. pylori* infection status** | | | | | | | |
| --- | --- | --- | --- | --- | --- | --- | --- | --- |
|  | Previously infected (n=124) | | Uninfected (n=27) | | Currently infected (n=42) | | Unknown (n=35) | |
| **Macroscopic type**, n (%) |  |  |  |  |  |  |  |  |
| Elevated | 19 | (15.3) | 6 | (22.2) | 10 | (23.8) | 8 | (22.9) |
| Flat or depressed | 105 | (84.7) | 21 | (77.8) | 32 | (76.2) | 27 | (77.1) |

**Online Resource 2.** Details of overall diagnosis performance of ME with 3G-NBI

| Endoscopic diagnosis | Pathological diagnosis | | | | Total |
| --- | --- | --- | --- | --- | --- |
|  | EGC (n=61) | | Non-EGC (n=167) | |  |
| Grade 1, n (%) | 7 | (7.7%) | 84 | (92.3%) | 91 |
| Grade 2, n (%) | 5 | (9.8) | 46 | (90.2) | 51 |
| Grade 3, n (%) | 6 | (25.0%) | 18 | (75.0%) | 24 |
| Grade 4, n (%) | 18 | (51.4) | 17 | (48.6) | 35 |
| Grade 5, n (%) | 25 | (92.6) | 2 | (7.4) | 27 |

ME, magnifying endoscopy; 3G-NBI, third-generation narrow-band imaging; EGC, early gastric cancer

**Online Resource 3.** Overall diagnosis performance of ME with 3G-NBI except gastric adenomas

A

|  |  | Pathological diagnosis, n | |
| --- | --- | --- | --- |
|  |  | EGC | Non-EGC |
| Endoscopic diagnosis | EGC | 43 | 16 |
|  | Non-EGC | 18 | 143 |

B

|  |  | |
| --- | --- | --- |
| Sensitivity, % (n/n) | 70.5 | (43/61) |
| Specificity, % (n/n) | 90.0 | (143/159) |
| PPV, % (n/n) | 72.9 | (43/59) |
| NPV, % (n/n) | 88.8 | (143/161) |
| Accuracy, % (n/n) | 84.5 | (186/220) |

**Online Resource 4.** Details of overall diagnosis performance of ME with 3G-NBI for gastric adenomas

| Endoscopic diagnosis | Pathological diagnosis |
| --- | --- |
|  | Gastric adenoma (n=8) |
| Grade 1, n | 1 |
| Grade 2, n | 1 |
| Grade 3, n | 3 |
| Grade 4, n | 2 |
| Grade 5, n | 1 |

ME, magnifying endoscopy; 3G-NBI, third-generation narrow-band imaging

|  | Overall (n=228) | | ***H. pylori* infection status** | | | | | | | |
| --- | --- | --- | --- | --- | --- | --- | --- | --- | --- | --- |
|  |  |  | Previously infected (n=124) | | Uninfected (n=27) | | Currently infected (n=42) | | Unknown (n=35) | |
| **Accuracy**, % (n/n) |  |  |  |  |  |  |  |  |  |  |
| Macroscopic type |  |  |  |  |  |  |  |  |  |  |
| Elevated | 93.0 | (40/43) | 100 | (19/19) | 100 | (6/6) | 90.0 | (9/10) | 75.0 | (6/8) |
| High confidence | 90.9 | (20/22) | 100 | (10/10) | 100 | (3/3) | 100 | (6/6) | 33.3 | (1/3) |
| Low confidence | 95.2 | (20/21) | 100 | (9/9) | 100 | (3/3) | 75.0 | (3/4) | 100 | (5/5) |
| Flat or depressed | 81.6 | (151/185) | 81.0 | (85/105) | 81.0 | (17/21) | 87.5 | (28/32) | 77.8 | (21/27) |
| High confidence | 92.7^***^ | (89/96) | 91.7^**^ | (55/60) | 88.9 | (8/9) | 100 | (15/15) | 91.7 | (11/12) |
| Low confidence | 69.7 | (62/89) | 66.7 | (30/45) | 75.0 | (9/12) | 76.5 | (13/17) | 66.7 | (10/15) |

**Online Resource 5.** Diagnostic accuracy of ME with 3G-NBI according to macroscopic type, confidence level, and *H. pylori* infection status

ME, magnifying endoscopy; 3G-NBI, third-generation narrow-band imaging

** P<0.01, *** P<0.001, No significant differences in other factors
